# Functional Synchronization of Biological Rhythms in a Tritrophic System

**DOI:** 10.1371/journal.pone.0011064

**Published:** 2010-06-10

**Authors:** Sufang Zhang, Jianing Wei, Xiaojiao Guo, Tong-Xian Liu, Le Kang

**Affiliations:** 1 State Key Laboratory of Integrated Management of Pest Insects and Rodents, Institute of Zoology, Chinese Academy of Sciences, Beijing, China; 2 College of Life Science, University of Science and Technology of China, Hefei, China; 3 Key Laboratory of Applied Entomology, Northwest A&F University, Yangling, China; Vanderbilt University, United States of America

## Abstract

In a tritrophic system formed by a plant, an herbivore and a natural enemy, each component has its own biological rhythm. However, the rhythm correlations among the three levels and the underlying mechanisms in any tritrophic system are largely unknown. Here, we report that the rhythms exhibited bidirectional correlations in a model tritrophic system involving a lima bean, a pea leafminer and a parasitoid. From the bottom-up perspective, the rhythm was initiated from herbivore feeding, which triggered the rhythms of volatile emissions; then the rhythmic pattern of parasitoid activities was affected, and these rhythms were synchronized by a light switch signal. Increased volatile concentration can enhance the intensity of parasitoid locomotion and oviposition only under light. From the top-down perspective, naive and oviposition-experienced parasitoids were able to utilize the different volatile rhythm information from the damaged plant to locate host leafminers respectively. Our results indicated that the three interacting organisms in this system can achieve rhythmic functional synchronization under a natural light-dark photoperiod, but not under constant light or darkness. These findings provide new insight into the rhythm synchronization of three key players that contribute to the utilization of light and chemical signals, and our results may be used as potential approaches for manipulating natural enemies.

## Introduction

The biological rhythm of most organisms is formed under an oscillatory environment of daily rotation of the planet earth, expressed as a roughly 24-hour cycle in their biochemical, physiological or behavioral processes [1]. These rhythms could be regulated by either endogenous circadian molecular machinery [2], [3] or external abiotic cues such as the presence or absence of light or food [4], [5]. Interestingly, it has also been suggested that biological factors, such as volatiles plants, can alter the behavior of an herbivore at different times of day [6], but this study did not provide clear evidence of rhythm interactions. Therefore, we sought to determine whether rhythms of different organisms could interact with each other, especially how abiotic and biotic factors could cooperatively regulate the rhythm interactions among different organisms. Here, we used a tritrophic system containing three closely related species to resolve this question.

In a tritrophic system, plants recruit natural enemies of herbivore insects to protect themselves from the damage of herbivores through herbivore-induced plant volatiles (HIPVs) [7], [8]. Each member in the tritrophic system exhibits its own behavioral and physiological rhythms [9], [10], [11], which can be affected by both biotic and abiotic factors. For example, in a tritrophic system containing a kidney bean, two-spotted spider mites and predatory mites, light is the dominant factor affecting HIPV production of bean plants, the feeding activity of herbivorous mites, and the predation of predatory mites [12]. In contrast, the volatiles from host plants, rather than light, play critical roles in affecting the nocturnal periodicity of the caterpillar *Mythimna separata* [6]. HIPVs display diurnal or nocturnal rhythms that may be coordinated with the habits of parasitoids that are attracted to them [8], [9], [13], [14]. The tritrophic interaction maintains its functional efficiency by synchronizing the rhythms across all trophic levels. If this synchronization is disrupted, the system may lose its stability [12]. However, the mechanisms underlying rhythm synchronization have not been elucidated in any tritrophic system. In particular, whether the coordination of tritrophic interactions is controlled by circadian clocks or by environmental abiotic and biotic factors is unknown. Tritrophic systems are good models for investigating the rhythm interactions among closely related species in a complex system and to explore the mechanisms underlying these interactions. In addition, it has been reported that parasitoids are able to learn and remember the chemicals from insect-damaged plants and to use these chemicals as cues to locate their hosts [15], [16]. However, how parasitoids respond to plant-emitted volatiles at different times of day has not been well characterized, and no studies have addressed the possibility that oviposition-experienced parasitoids respond specifically to infochemicals emitted at a particular time of day. On the other hand, chemical ecology studies on different tritrophic systems have yielded promising control strategies for certain crop pests [17], [18]. Therefore, research on biological rhythm interactions in a tritrophic system is not only of theoretical significance as a mechanism for plant-insect-parasitoid interactions and rhythm biology, but also a potential guidance for developing new pest management strategies.

In this study, we selected a widely used model tritrophic system consisting of a plant *Phaseolus lunatus* (lima bean), a pest *Liriomyza huidobrensis* (pea leafminer) and a natural enemy of the pest *Opius dissitus* (parasitoid) to reveal the rhythm synchronization and the factors affecting it. We demonstrate that the rhythms of each member in this system were strongly synchronized by light. Initiated by the feeding of leafminers, the chain of events is followed by the plant volatile emissions, and then the parasitoid activities. All of these interactions were examined under different light and dark cycles. In contrast to the clear periodicity across all three trophic levels under LD cycles, the synchronization of behavioral rhythms was lost under constant light or darkness (LL or DD). Furthermore, unlike the naive parasitoids, Y-tube behavior experiments indicate that oviposition-experienced parasitoids were able to discriminate volatiles emitted from damaged plants at different time intervals during the day. Taken together, our results provide solid evidence that the three key players in the tritrophic system have co-evolved under an LD cycle in nature to achieve a functional synchronization of their diurnal rhythms.

## Results

### Rhythms of the tritrophic system under LD (15∶9) cycle

**Figure 1:**
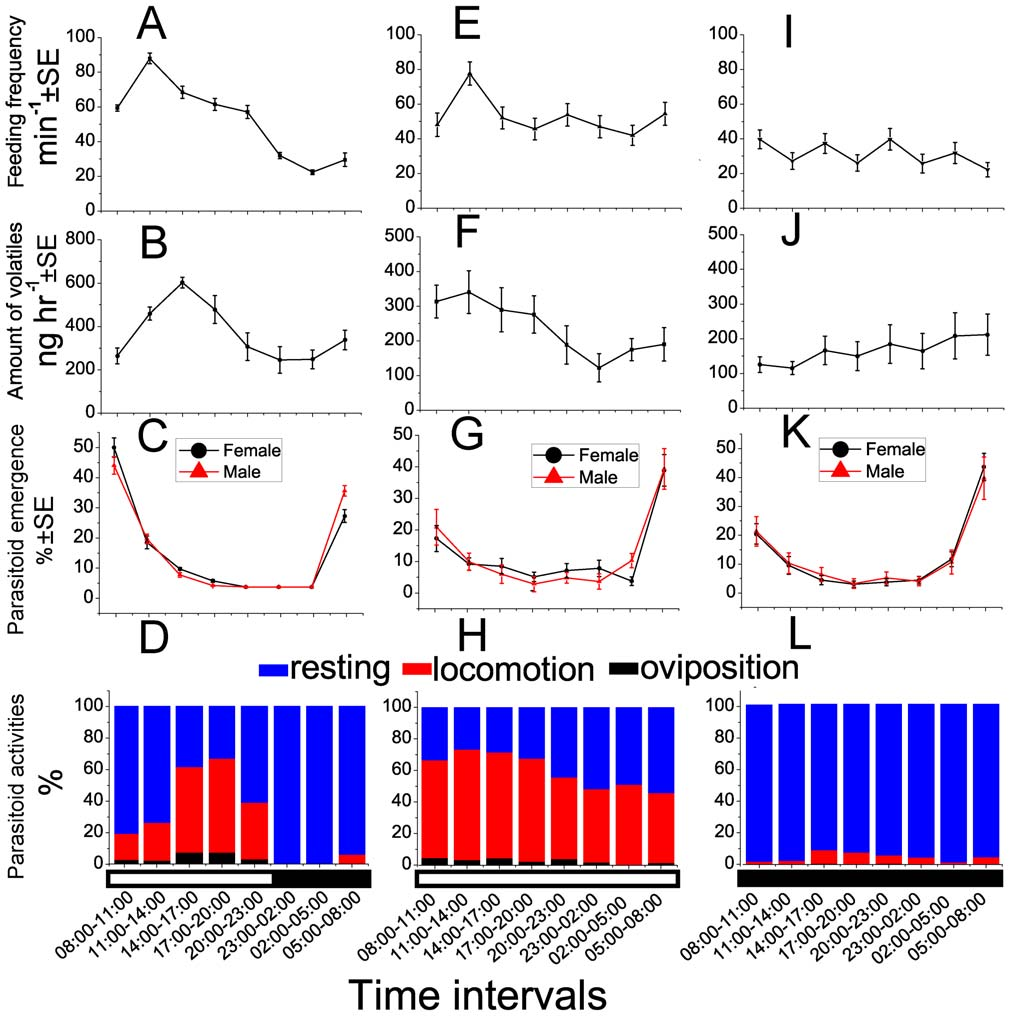
Diurnal rhythm of three trophic levels in the tritrophic system under three photoperiods. Four parameters were obtained: leafminer larval feeding frequency (mean±SE, n>30), absolute amount of whole volatiles collected from the leafminer-damaged lima bean plants per 3-h interval (mean±SE, n = 6), relative emergence (mean±SE, n>10), and activities of *O. dissitius* (n = 12). (A)–(D), diurnal rhythms under LD photoperiod. (E)–(H)**,** diurnal rhythms under LL photoperiod. (I)–(L)**,** diurnal rhythms under DD photoperiod. The bars under the x axis refer to the photoperiodic cycle: the white part refers to the light phase; the black part refers to the dark phase.

**Figure 2:**
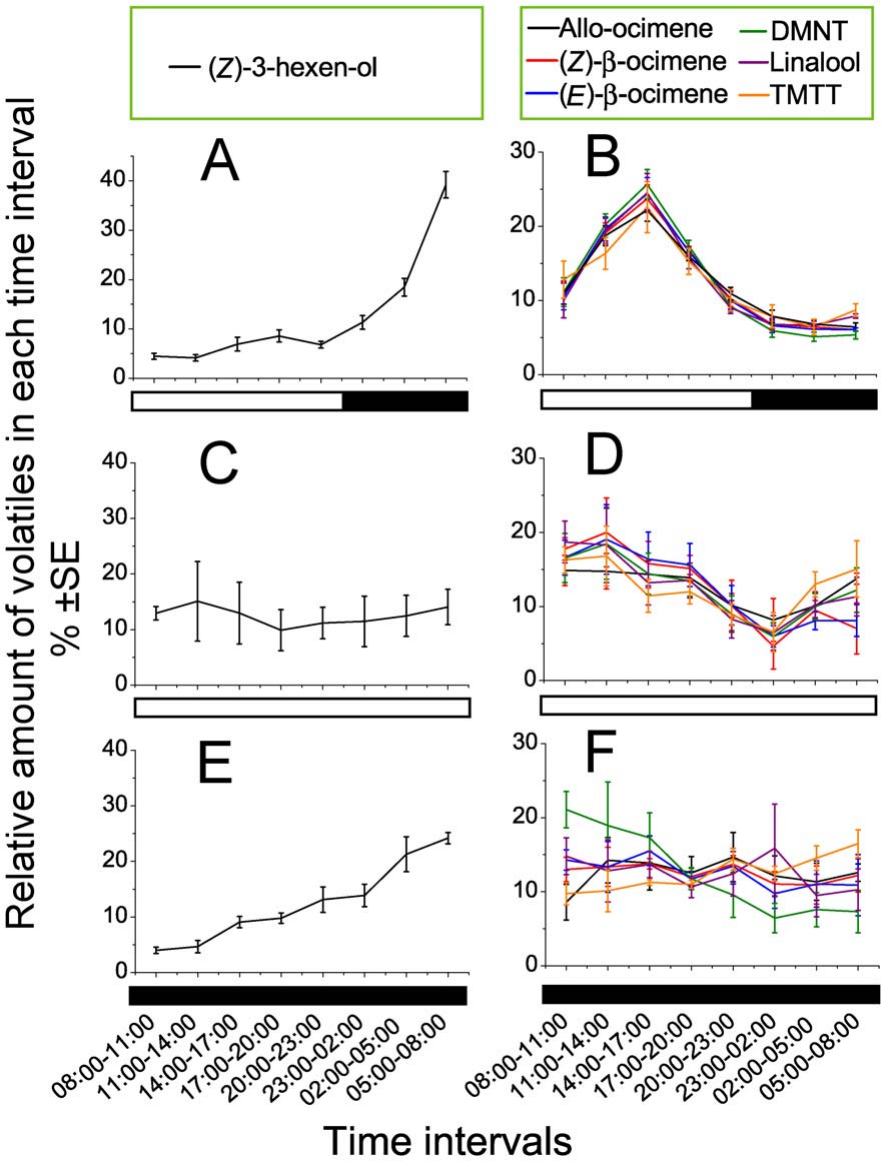
Diurnal rhythms of two typical kinds of volatiles from leafminer-damaged plants under three photoperiods. Rhythms of the relative amounts of (*Z*)-3-hexen-ol (A) and terpenes (B) under LD photoperiod (mean±SE, n = 6); rhythms of relative amounts of (*Z*)-3-hexen-ol (C) and terpenes (D) under LL photoperiod (mean±SE, n = 6); rhythms of relative amounts of (*Z*)-3-hexen-ol (E) and terpenes (F) under DD photoperiod (mean±SE, n = 6). The bars under the x axis refer to the photoperiodic cycle: the white part refers to the light phase; the black part refers to the dark phase. DMNT: (3*E*)-4,8-dimethyl-1,3,7-nonatriene; TMTT: (3*E*,7*E*)-4,8,12-trimethyl-1,3,7,11-tridecatetraene.

Most tested items in the tritrophic interaction exhibit obvious, closely correlated rhythms under LD (15∶9), which is the typical photophase during the growth seasons in tropical and temperate regions. The feeding activity of second instar pea leafminers, expressed as the oral hook moving frequency, displayed high variance during the diurnal cycle, and it was more evident during the photophase than during the dark phase (Figure 1A). Leafminer larvae feeding frequency increased from 08:00 to 11:00, peaked from 11:00 to 14:00, then slowly decreased and reached its lowest level from 03:00 to 04:00 during the dark phase, and gradually increased at the beginning of the next diurnal cycle (*F*_7, 200_ = 68.566, *P = *9.5E-50). As few volatiles were detected from uninfected lima bean plants (Figure S1A), we focused on those emitted from plants infected by leafminers (Figure S1B). The amounts of different volatiles varied greatly during the diurnal cycle (*F*_14,75_ = 35.1735, *P* = 3.2E-27), and most inducible volatiles were emitted with distinct diurnal rhythms (Figure S2). Total volatile emission started to increase at the beginning of the photophase and peaked between 14:00 and 17:00, gradually decreased afterward, and remained at low levels between 23:00 and 05:00 during the dark phase (*F*_7,40_ = 7.355, *P* = 1.1E-05) (Figure 1B). We found that the dominant volatiles can be divided into two main categories based on their different rhythms. Starting at low levels, the emission of (*Z*)-3-hexen-ol slowly increased from the beginning of the photophase, and peaked between 05:00 and 08:00 in the dark phase (Figure 2A). The emission rhythms of terpenoids, including (*Z*)-b-ocimene, (*E*)-b-ocimene, allo-ocimene, linalool, (3*E*)-4,8-dimethyl-1,3,7-nonatriene (DMNT) and (3*E*,7*E*)-4,8,12-trimethyl-1,3,7,11-tridecatetraene (TMTT), peaked between 14:00 and 17:00 and corresponded well with the rhythm of total volatiles (Figure 2B). Furthermore, we also measured the rhythms of parasitoid emergence, locomotion, and oviposition. Emergence of the parasitoid adult began at 05:00–08:00 in anticipation of photophase, and peaked at early photophase between 08:00 and 11:00 (Female: *F*_7, 64_ = 29.291, *P* = 1.1E-17; Male: *F*_7, 64_ = 30.970, *P* = 3E-18) (Figure 1C), with the majority of parasitoids (∼80%) emerging before 11:00, regardless of the sex. Locomotion and oviposition activity displayed similar rhythms (Figure 1D). During the photophase, the parasitoids gradually became more active, and locomotion and oviposition activities peaked from 14:00–19:00; thereafter, they gradually became inactive and almost quiescent during the dark phase. The time that the parasitoids spent in locomotion was 7-fold longer than time spent in oviposition (locomotion: *F*_7, 88_ = 45.355, *P* =  1.5E-26; oviposition: *F*_7, 88_ = 7.451, *P* =  5.1E-07) (Figure 1D).

The tritrophic interactions of larval feeding, volatile emissions, and parasitoid activities were sequentially correlated under LD (Table S1). Larval feeding-induced lima bean volatile emissions could mainly be divided into two categories: terpenes increased after the light was on and peaked in afternoon; while fatty acid-derived volatiles increased immediately when the larvae began feeding in early morning, and peaked in anticipation of photophase (Table S2). Most volatiles were emitted a few hours after larval feeding, suggesting that the leafminer feeding rhythm did not develop in response to plant volatile emissions; instead, volatiles were induced by the feeding of leafminers. Parasitoid locomotion and oviposition rhythms were closely correlated with the emission rhythm of terpenes; intriguingly, the parasitoid emergence rhythm was more synchronized with (*Z*)-3-hexen-ol (r_Parasitoid emergence-(*Z*)-3-hexen-ol_ = 0.7870; *P* = 0.02042), with the peak delayed by one time interval. From these data, we can conclude that the rhythms of three key players in the tritrhophic interaction were closely correlated to each other. However, whether these correlations are genetically controlled or can be affected by light was still unclear.

### Rhythms of the tritrophic system under constant light (LL) and darkness (DD)

To further determine whether the rhythms of three key players in the tritrohpic system were controlled by the endogenous circadian clocks or induced by photoperiod, we measured the rhythm profiles under constant light (LL) and darkness (DD), respectively. Under LL, the leafminer larval feeding remained in similar rhythm, without a significant decrease from 23:00–08:00 as observed in LD (Figure 1E), while leafminer larval feeding activity displayed an arrhythmic pattern under DD (Figure 1I). Total volatile emission under LL showed a weak rhythm, which was similar to the pattern under LD, except that the phase was shifted ahead three hours (Figure 1F). The terpenes showed similar dampened rhythms to the total volatiles (Figure 2D), but (*Z*)-3-hexen-ol became arrhythmic (Figure 2C). Under DD, all volatile emissions except (*Z*)-3-hexen-ol became arrhythmic and in low concentration (Figure 1J, 2E, 2F, Figure S2). Parasitoid emergence rhythms showed similar patterns in both LL and DD as in LD (Figure 1G, 1K), indicating that this rhythm was regulated more by genetic circadian clock machinery rather than by light. Parasitoids spent most time in locomotion under LL, with a small portion for oviposition (Figure 1H). However, under DD, the fluctuations of parasitoid locomotion or ovipositional activity were very slight (Figure 1L), suggesting that these parasitoid activities are diurnal, and their activity is stimulated by light and suppressed by darkness.

Taken together, the rhythm correlations of plant volatile emissions, larval feeding, and parasitoid activities were disrupted under LL and DD (Table S1). Under LL, all volatiles emitted after leafminer larval feeding showed similar weak rhythms and were well correlated with each other; the rhythm of leafminer feeding was still correlated to these volatiles. Parasitoid oviposition well matched these volatiles, but parasitoid locomotion did not match the volatile emission rhythms from the plants (Table S3). Under DD, there was little consistency among the various rhythms in the tritrophic system (Table S4). Experiments under LL and DD indicated that the rhythms in the trithophic interaction were dramatically affected by light. However, the locomotion and oviposition of parasitoids were still rhythmic under LL, suggesting that light was not the only controlling factor.

### Parasitoid activities under artificially enhanced volatile concentration

**Figure 3:**
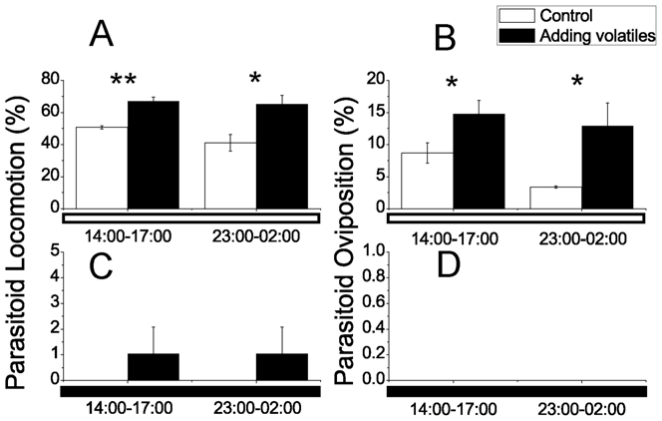
Changes in parasitoid locomotion and oviposition after artificially adding the plant volatile concentrations. (A), Parasitoid locomotion change after adding plant volatile concentrations during two time intervals under light (mean±SE, n = 4) (two-tailed test, 14:00–17:00: *F*_1,4_ = 32.820, *P* = 0.0046; 23:00–02:00: *F*_1,4_ = 9.186, *P* = 0.0351); (B), Parasitoid oviposition change after adding plant volatile concentrations during two time intervals under light (mean±SE, n = 4) (two-tailed test, 14:00–17:00: *F*_1,4_ = 7.296, *P* = 0.043; 23:00–02:00: *F*_1,4_ = 16.41, *P* = 0.015); (C), Parasitoid locomotion change after adding plant volatile concentrations during two time intervals under dark (mean±SE, n = 5); (D), Parasitoid oviposition change after adding plant volatile concentrations during two time intervals under dark (mean±SE, n = 5). *P<0.05; **P<0.01. The bars under the x axis refer to the photoperiodic cycle: the white part refers to the light phase; the black part refers to the dark phase.

To investigate whether plant volatiles or light regulates the phase of the parasitoid locomotion and oviposition rhythms, we manipulated the plant volatiles by releasing a high concentration in the chamber under LL and DD, respectively, and determined parasitoid locomotion and oviposition at two different time intervals—14:00–17:00 in the afternoon and 23:00–02:00 during the night. We found that locomotion and oviposition of the parasitoids were elevated by the increased volatile concentrations at both time intervals under LL (14:00–17:00: locomotion, *F*_1,4_ = 32.82, *P* = 0.0046, oviposition, *F*_1,4_ = 7.296, *P* = 0.043; 23:00–02:00: locomotion, *F*_1,4_ = 9.816, *P* = 0.0351, oviposition, *F*_1,4_ = 16.41, *P* = 0.015)(Figure 3A, 3B). However, enhanced volatile concentration did not promote the intensity of parasitoids locomotion and oviposition under DD (Figure 3C, 3D). Therefore, light is a switch of parasitoid activities, whose phase can be affected by volatile concentration under light.

### Different olfactory responses of naive and oviposition-experienced parasitoids

**Figure 4:**
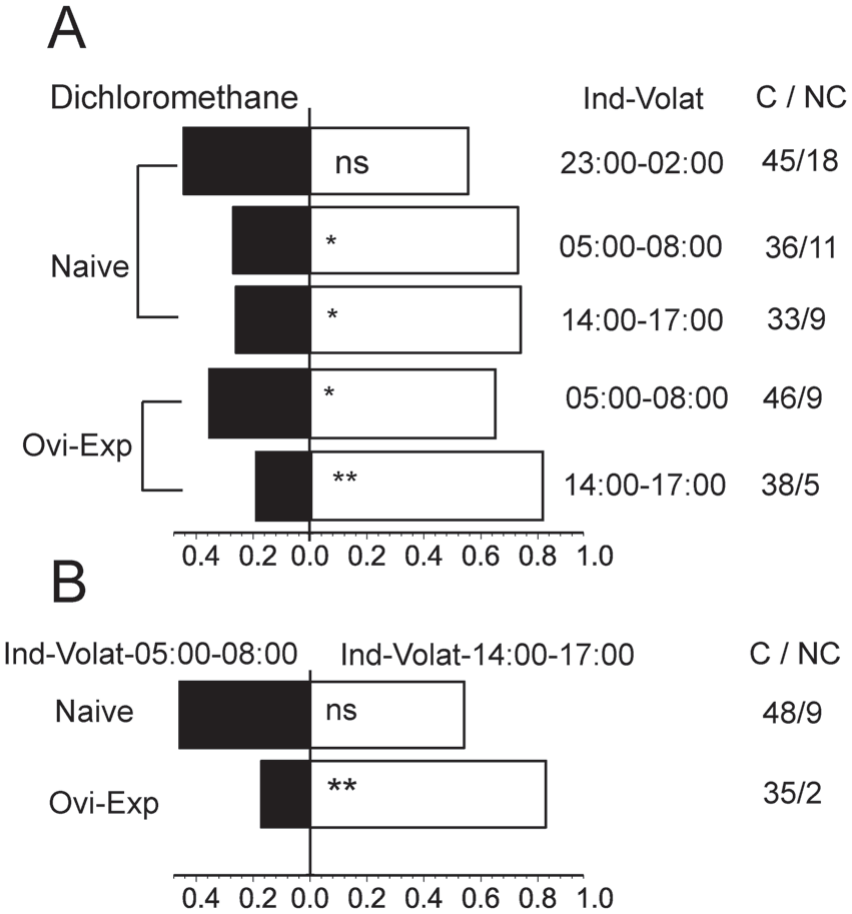
Behavioral responses of naive and oviposition-experienced *O. dissitius* adults to volatiles. The volatiles were extracted from leafminer-damaged lima beans during selected time intervals: terpenoids-dominated blend from 14:00–17:00, fatty acid-derived chemical-abundant blend from 5:00–8:00, and volatile-deficient blend from 23:00–02:00: (A), the response of naive and oviposition-experienced parasitoids to a single volatile extraction (naive parasitoid: 14:00–17:00, χ^2^ = 5.261, *P* = 0.0218; 05:00–08:00, χ^2^ = 5.538, *P* = 0.0186; 23:00–02:00, χ^2^ = 0.556, *P* = 0.456. Oviposition-experienced parasitoids: 05:00–08:00, χ^2^ = 4.261, *P* = 0.039; 14:00–17:00, χ^2^ = 15.158, *P*<0.0001); (B), the response of naive and oviposition-experienced parasitoids to two volatile blends, blend from 5:00–8:00 and blend from 14:00–17:00 (naive parasitoids: χ^2^ = 0.333, *P* = 0.564; oviposition-experienced parasitoids: χ^2^ = 15.114, *P* = 0.0001). χ^2^ test was used to test for significant differences between numbers of parasitoids in each arm. *P<0.05; **P<0.01. More than 30 females made choices in each experiment. C/NC: Choice/No Choice; Ovi-Exp: Oviposition experienced.

Since emissions of (*Z*)-3-hexen-ol and terpenes were dominant in the morning and afternoon, respectively, and naive parasitoid emergence and oviposition-experienced parasitoid activity exhibited similar patterns associated with these two chemicals, we tested whether the olfactory responses of naive and oviposition-experienced parasitoids correlate with the emission patterns of these volatiles. A Y-tube olfactometer was used to examine the behavioral responses of naive and oviposition-experienced parasitoids to the volatile extracts emitted at different time intervals under the normal LD cycle: during the dark phase (23:00–02:00) when all the chemicals were weak, between the dark and light cycles (05:00–08:00) when (*Z*)-3-hexen-ol characterized fatty acid-derived volatiles were dominant, and during the light photophase (14:00–17:00) when terpenes were dominant. In the first experiment (Figure 4A), naive and oviposition-experienced parasitoids were offered a choice between the control volatile dichloromethane and volatile blends emitted at three different time points. Naive female parasitoids showed a significant preference for volatile blends emitted during the two time intervals, 14:00–17:00 (χ^2^ = 5.261, *P* = 0.0218) and 05:00–08:00 (χ^2^ = 5.538, *P* = 0.0186), but not for the volatile blend emitted from 23:00–02:00 (χ^2^ = 0.556, *P* = 0.456). Oviposition-experienced parasitoids preferred the volatile blend emitted from 05:00–08:00 (χ^2^ = 4.261, *P* = 0.039); however, the volatile blend emitted from 14:00–17:00 more obviously attracted oviposition-experienced parasitoids (χ^2^ = 15.158, *P*<0.0001). In the second experiment (Figure 4B), naive and oviposition-experienced parasitoids were exposed to the volatiles normally emitted at 05:00–08:00 and 14:00–17:00 simultaneously. The naive female parasitoids did not display significant preference for either of the two volatile blends (χ^2^ = 0.333, *P* = 0.564). However, oviposition-experienced parasitoids preferred the volatiles emitted from 14:00–17:00 to those emitted from 05:00–08:00 (χ^2^ = 15.114, *P*<0.0001).

## Discussion

In this study, we investigated the rhythms in a tritrophic system under LD, LL and DD, and presented a clear illustration of the rhythm correlations among three different organisms. It seems that they exhibited bidirectional correlations. From the bottom-up perspective, the results indicate that the biological rhythms of the lima bean, leafminer and parasitoid system are synchronized under the natural light-dark cycle, and the correlation was initiated from herbivore feeding, followed by the rhythms of volatile emissions, and then the rhythms of parasitoid activities. Our data showed that healthy lima beans produce few volatiles, but when damaged by leafminers, lima beans emitted a large amount of volatiles, and the larval feeding rhythm paralleled that of terpene emission from the leafminer-damaged lima bean plants but with one interval (3 hours) ahead. These observations suggest that volatiles were induced by the feeding of leafminers, and it took several hours for plants to respond to the damages from herbivores, as has been described before [9]. Parasitoid locomotion corresponded well with the emission of terpenes, and manipulating the plant volatiles by providing a high concentration in the chamber increased both parasitoid locomotion and oviposition rates (Figure 3A, 3B). Thus, the volatile emission rhythms drove the periodic activities of the parasitoids. However, the synchronized rhythms across the tritrophic levels were disrupted under LL and almost disappeared in the DD condition, indicating that light was a necessary synchronizer in this system. From the top-down perspective, the naive and oviposition-experienced parasitoids can differently interpret the volatile rhythm information, which may help the parasitoid locate its host efficiently.

The feeding frequency of the leafminer larvae had a well defined rhythm under LD or LL, and the rhythm largely disappeared in the DD condition, indicating that the feeding rhythm of leafminer larvae is entrained by light. The volatile emission rhythms followed the rhythms of larvae feeding, which proved that the volatiles result from larvae feeding in a rhythmic way. Our results differ from the reports by Shiojiri *et al* [6], in which light has no impact on driving the rhythm of herbivore activity, but plant volatiles activate herbivore behavior. Because our and Shiojiri's experiments were performed with different tritrophic systems, this difference suggests that the coevolution of various tritrophic systems resulted in distinct interaction models.

Furthermore, the emission of plant volatiles was also affected by the lighting condition, and more interesting, different classes of volatiles have different emission rhythms, even in the same plant-herbivore system. Our analyses focused on the volatiles that were emitted in relatively higher levels and were more attractive to parasitoids [20], such as (*Z*)-3-hexen-ol and terpenes. Under the LD cycle, terpenes were mainly released in the photophase, while fatty acid-derived volatiles, especially (*Z*)-3-hexen-ol, accumulated during the night. Under the LL condition, the emission rhythm for terpenes remained similar, but the concentration of fatty acid-derived volatiles was extremely low and non-rhythmic. In contrast, under the DD condition, the fatty acid-derived volatiles gradually accumulated over time, but the terpene release was low. Thus, fatty acid-derived volatiles, especially (*Z*)-3-hexen-ol, and terpenes had different responses to light, and their emission patterns were opposite.

The stable rhythm of parasitoid emergence under various photoperiod cycles reveals that this behavior is controlled by an internal circadian clock. The emergence of naive parasitoids in the morning was significant for parasitoids to locate their host habitat, which will be explained later. Parasitoid locomotion and oviposition, on the other hand, were active under LL or the light phase of LD, but were nearly quiescent under DD or the dark phase of LD (Figure 1D,1H,1L), indicating that these two behaviors were highly associated with light. This conclusion is supported by observations in other systems [21]. In our study, parasitoid locomotion and oviposition were also correlated with the emission of terpenes. When we increased the concentration of plant volatiles in the chamber at different time points under light, both locomotion and oviposition rates of parasitoids were significantly augmented (Figure 3A, 3B). In darkness, the parasitoids remained inactive in the same volatile concentration (Figure 3C, 3D). Thus, we can conclude that the rhythms of the parasitoid locomotion and ovipositon were diurnal, but the activity can be influenced by light and volatile concentrations. The former acts as a switch signal for parasitoid locomotion and oviposition, and the latter modulates the intensity of parasitoid activity.

As observed in our system, the volatile emission rhythm is a result of the herbivore feeding rhythm, and then the parasitoids are rhythmically recruited according to the concentration of plant volatiles, suggesting that organisms are also able to actively adjust their biological rhythms according to those of other organisms, which could be described as a social synchronization of rhythms [22].

Our Y-tube behavior experiments demonstrate that oviposition-experienced parasitoids can learn to use the rhythm information from volatiles, and were more sensitive to terpene-dominated volatiles, which were an accurate indication of their host insect. The peak of parasitoid emergence coincided with the peak of (*Z*)-3-hexen-ol emission, and naive parasitoids showed a strong preference for (*Z*)-3-hexen-ol in behavioral experiments (Figure 4A). As a small chemical molecule, (*Z*)-3-hexen-ol can be released at high concentrations and can spread across a long distance [23], [24]. Parasitoid pupae often fall into the soil surface, and after emergence, the first thing the adults should do is locate their host habitat, before searching for their host insects [25]. (*Z*)-3-hexen-ol's stronger attraction to naive parasitoids could be beneficial for the parasitoids to locate their host habitat from a longer distance [23], because the new emergence of naive parasitoid and the (*Z*)-3-hexen-ol emission both peaked in the morning. However, (*Z*)-3-hexen-ol can be induced both when plants are damaged by insects or by other mechanical factors [26], [27], and terpenes are more associated with insect damage [28]. In this regard, terpenes are more accurate signals for parasitoids to locate host. It is not surprising that oviposition-experienced parasitoids, having already located hosts, would have a stronger response to the potential and accurate oviposition stimulant terpenes than to a host habitat location signal (*Z*)-3-hexen-ol. Thus, naive and experienced parasitoids differently interpret the plant volatiles emission rhythms to help themselves find their hosts most efficiently.

In summary, our results indicate that the synchronization of biological rhythms in the tritrophic interactions were bidirectional: firstly, the rhythm of leafminer larval feeding drives the oscillating emissions of plant inducible volatiles, which in turn determines the rhythm of parasitoid oviposition and locomotion, and they are synchronized by light-dark cycle; secondly, parasitoids can take advantage of the inherent information of volatiles emitted from the damaged plant at different times to locate their leafminer host by experience-guided learning. It appears that the three key players in the tritrophic system have co-evolved rhythms as well as functional synchronization under the natural light-dark cycle, which are achieved through a combination of programmed circadian rhythms, light-affected diurnal rhythms, directly induced responses and experience-guided learning. Biological rhythm synchronization is a common phenomenon in nature; it can occur among inner-species [22], [29], [30], [31] or in a system consisting of different species as shown in our research, and it is beneficial for reproduction or protection [22]. In our system, the synchronization of plant volatile emissions and parasitoid activities could facilitate both the protection of the plant and the host location of parasitoids, which may explain the efficient indirect defense of the tritrophic system in nature [26], [32]. Our findings could also help implement biorational pest management programs in various agroecosystems [17], [18].

## Materials and Methods

### Environmental Conditions and Photophases

The environmental conditions inside the chambers were set at 25±1°C, 60±10% RH; and light intensity of 12.65 W/m^2^ during photophase. Three different photoperiodic conditions were used: 15-h light: 9-h dark (LD), constant light (LL), and constant darkness (DD); experiments under LL and DD were performed after one day adaption in the same condition. The insect behavior during the dark phase was monitored under red light (620 nm – 690 nm, 4 W/m^2^) to avoid disturbance.

### Plants and Insects

Lima bean plants (*Phaseolus lunatus* L. cv Sieva) were individually seeded in plastic pots (12 cm in diameter) with a mixed potting medium of peat and vermiculite (3∶1) in the environmental chambers. The bean plants with two fully-developed true leaves were used in all experiments.

Pea leafminers (*Liriomyza huidobrensis*) and the parasitoids (*Opius dissitus*) have been cultured under laboratory conditions for three years. The lima bean was used as the host plant of *L. huidobrensis*, which was then used as the host for rearing the parasitoid.

To characterize larval feeding, mated *L. huidobrensis* adults were released on lima bean leaves for oviposition. The total number of adult leafminers was about 300. Before the experiment, the adults were fed only 10% diluent honey for 10 h, and then the lima bean plant was placed into the cage containing adult leafminers. We tried to keep the leafminer number constant in each replicate of the experiment. The adults were removed within 4 h. The second instar larvae (96 h after oviposition) were used for studying feeding activity in lima bean leaves.

Both naive and oviposition-experienced *O. dissitus* adults were used. The naive parasitoids were 1–2 day old adult females without previous exposure to their host or host plants. The oviposition-experienced parasitoids were 3–4 day old adult females that have oviposited on the second instar leafminer larvae fed on lima bean leaves for one day; then they were collected again and used in experiments within 5 h.

### Diurnal feeding rhythm of leafminer larvae

The feeding rhythm of leafminer larvae was expressed as the frequency of oral hook movements per minute inside a leaf. One diurnal cycle was divided into eight 3-h time intervals, and we examined the feeding frequency in the middle hour of each time interval. One larva was counted for one minute under a stereomicroscope (Wild, Heerbrugg, Switzerland), the observation was made on more than six leaves, and 8–10 larvae were selected from each leaf. The experiments were replicated more than twelve times. For experiments under LL and DD, the plants was grown under LD for two weeks, and the plants with two fully-developed true leaves was fed to leafminers for 4 h; these leafminer-damaged plants were further grown under LD for 48 h and then transferred into LL or DD. After 24 h, the feeding rhythm was measured as described above. The counting under DD was performed under red light to avoid the disturbance of white light. The larva number in each plant was 200–300, and this number was kept constant each time the experiment was repeated.

### Volatile collection

There were three treatments: (1) clean bags - the odor collected from a clean oven bag (Reynolds Oven Bags, Reynolds Kitchens, Richmond, VA, USA) was used as a control; (2) healthy bean; and (3) damaged plants - lima beans that were damaged by second instar leafminer (96 h after oviposition).

The headspace volatile collection setup was prepared as previously described [19]. To measure the diurnal cycle of plant volatile emissions, one diurnal cycle was divided into 8 time intervals as in the larvae feeding experiments, and the volatiles were collected 8 times in the 3-h intervals starting from 08:00. Experiments under LL and DD were performed for two consecutive days, with the first day as an adaption day. The plants used under LL and DD were similarly treated as described in the leafminer observation experiments. They were grown and damaged by leafminers, and transferred to LL or DD for volatile extraction. The larva in each lima bean plant was about 200–300 as in larvae feeding experiments, and this number was kept constant each time the experiment was repeated. The absorbing glass collector connected to each bag was replaced with a new one for each time interval, and the collector was changed under a red light in the experiments performed in DD. All aeration extracts were stored at −20°C until used in chemical analyses or behavioral experiments. The plants were weighed immediately after collection and the number of leafminer larvae on the leaves was recorded.

### Chemical identification and quantification

Collected volatiles were identified as described by Wei et al [19]. A GC (7890A; Agilent Technologies, Inc., Santa Clara, CA, USA) coupled with an auto-inlet installation was used to quantify the collected volatiles. The system was equipped with the same DB-WAX column and the same template program as the previous GC-MS system. Heptanoic acid, ethyl ester and dodecanoic acid, ethyl ester (1 ng/µL^1^, 5 ng/µL^1^, 20 ng/µL^1^, 50 ng/µL^1^ and 100 ng/µL^1^) were used to develop standard curves to quantify volatiles in the samples.

### Diurnal rhythm activities of the parasitoids

#### Emergence

One hundred pupae of *O. dissitus* were placed in a glass tube in an environmental chamber. The emergence rhythm of *O. dissitus* was monitored at the same time interval as volatile collections. The emerged females and males were counted and collected in a glass tube (70×8 mm) in each time interval for five consecutive days. Then the number of parasitoids that emerged at the same time interval over the five days was added together. The relative emergence rhythm of the parasitoid in one day was calculated. Each experiment was replicated more than 10 times. For investigations under LL and DD, the pupae were collected from lima beans grown under LD, but the newly collected pupae were placed under LL or DD. After about 10 days, the parasitoid began to emerge and the rhythms were monitored under LL or DD. The measurement under DD was performed under a red light.

#### Oviposition, locomotion and resting of female parasitoids

Three lima bean plants damaged by leafminer larvae were put in a glass jar (300×300×350 mm) and 15 *O. dissitus* female adults were released in each jar. The ovipositions, locomotor and resting of parasitoids were recorded for 10 minutes in each hour, 3 times in each 3-h interval. The observation was repeated more then 6 times under each light condition. For experiments under LL or DD, the parasitoids were brought into LL or DD for one day adaption, and then measured under the same light condition. The measurements under DD were performed under red light to avoid white light disturbance.

### Parasitoid locomotion, oviposition and resting with artificially supplemented volatiles at particular time points

To investigate the impact of volatiles on parasitoid activities, we artificially added the volatiles to the jar in a concentration that matched the peak volatile levels in the LD cycle at two time points: 14:00–17:00, when the plants emitted large amounts of volatiles and the parasitoids were active; 23:00–02:00, when plants emitted few volatiles and the parasitoids were inactive. The peak volatile emission rate is about 600 ng hr^−1^ (Figure 1B) under LD. For the time interval of 14:00–17:00 under LL, the volatile emission rate is about 300 ng hr^−1^ (Figure 1F), thus 1/2 volume of the volatiles collected under LD during 14:00–17:00 was used; For the time interval of 23:00–02:00 under LL, 14:00–17:00 under DD, and 23:00–02:00 under DD, the volatile rate is about 100 ng hr^−1^ (Figure 1F, 1J), thus 5/6 volume of the volatiles collected under LD during 14:00–17:00 was used. The volatiles were added to a cotton ball fixed to the top of the jar and then the jar was sealed. After adding the volatiles, we monitored the locomotion and oviposition of the parasitoids as described above. The control experiments were performed using the solvent of volatiles. The experiments under LL and DD cycle were repeated more than 3 times each. The parameters under DD were measured under red light.

### Olfactory responses of the naive and oviposition-experienced parasitoids to volatiles emitted at different time points

The experiment was performed as described by Wei and Kang [20]. A Y-tube olfactometer was used to examine the olfactory and behavioral responses of naive and oviposition-experienced parasitoids to the volatile extracts of early morning and mid-afternoon, respectively. The Y-tube olfactometer (stem, 10 cm; arms, 23 cm at 60° angle; internal diameter 2.3 cm) was put into an observation chamber (95×60×45 cm). On top of the device, there were four 22-W cool, white fluorescent tubes, which can provide uniform lighting. Through air conditioning, the temperature in the chamber was maintained at 26±1°C. Air was purified and humidified through activated charcoal and a water jar, and then a pump (Beijing Institute of Labour Instruments, China) was used to draw the air into the Y-tube. By a flowmeter, the airflow through each of the olfactometer arms was maintained at 250 ml/min. There was a piece of filter paper (1×2 cm) in each arm of the Y-tube olfactometer; the volatile extracts or the control chemicals were put on the paper and set into the arms. New filter papers with the extracts or control chemicals were used for each parasitoid. After tests of 5 individuals, the position of the arms containing treatment and control odors was reversed to avoid position bias. After tests of 10 individuals, the Y-tube was replaced. Each female parasitoid was released in the olfactometer for 5 min. If the parasitoid moved more than 5 cm into either arm, we recorded this as “first choice.” If the parasitoid was inactive within the experimental duration, we recorded as “no choice.”

Two experiments were performed. In the first experiment, the naive and oviposition-experienced parasitoids were offered a choice between the solvent control dichloromethane or other selected volatiles. Volatile extract was applied to a piece of filter paper (1×2 cm) at a volume of 10 microliters and was placed inside one arm of the Y-tube olfactometer. A same-size filter paper impregnated with an equal volume of HPLC-grade dichloromethane was set in the other arm as a control. In the second test, the naive and oviposition-experienced parasitoids were offered a choice between the inducible volatiles collected at two different time intervals (05:00–08:00 and 14:00–17:00), and the two extracts were applied to the two arms, respectively. Each parasitoid was used only once. A minimum of 35 females that made a choice were examined in each experiment.

### Data analysis

The data were analyzed using the analysis of variance (ANOVA), and means were separated using Tukey's significant difference (HSD) test (SPSS 15.0; SPSS Inc., Chicago, Illinois, USA). Student's *t*-test or ANOVA followed by Tukey's HSD test were used to compare volatile emissions from different plant treatments. A χ^2^ test was used to determine the significance of the differences in the bioassay of parasitoid olfactory behaviors in the olfactometer [20]. The parasitoids that did not make a choice were excluded from statistical analyses. Unsuccessful parasitoid response rates in choice experiments ranged from 10 to 25%. Pearson's correlation was used to determine the correlations between the leafminer feeding, emission of plant volatiles, and parasitoid emergence, locomotion, and oviposition in diurnal cycles.

## Supporting Information

Figure S1 Headspace sample analysis with GC (7890A; Agilent Technologies, Inc., Santa Clara, CA, USA). (A), Headspace sample of healthy lima bean plant. (B), Headspace sample of 2 instars leafminer infected lima bean plant. Time intervals in the figure indicate the collection time interval of the headspace sample.
(0.59 MB TIF)

Figure S2 Diurnal rhythms of absolute amount of fourteen detected volatiles under three conditions (mean ± SE, n = 6). The bar under the x axis in each figure refers to the photoperiodic cycle; the black part refers to the dark phase. DMNT: (3E)-4,8-dimethyl-1,3,7-nonatriene; TMTT: (3E,7E)-4,8,12-trimethyl-1,3,7,11-tridecatetraene.
(2.90 MB PDF)

Table S1 The most significant correlations among leafminer feeding, inducible emission and parasitoid activity (Pearson correlation at 0.01 level).
(0.07 MB DOC)

Table S2 Pearson Correlation analysis of the tritrophic interaction rhythms under LD (L∶D = 15∶9) cycle (Pearson correlation at 0.01 level).
(0.12 MB DOC)

Table S3 Pearson Correlation analysis of the tritrophic interaction rhythms under constant light (LL) cycle (Pearson correlation at 0.01 level).
(0.12 MB DOC)

Table S4 Pearson Correlation analysis of the tritrophic interaction rhythms under constant dark (DD) cycle (Pearson correlation at 0.01 level).
(0.12 MB DOC)
