# Supplementary figures and images for: Functional Synchronization of Biological Rhythms in a Tritrophic System
Source: PLoS One. 2010 Jun 10;5(6):e11064. doi: 10.1371/journal.pone.0011064 (PMC2883855; doi:10.1371/journal.pone.0011064)

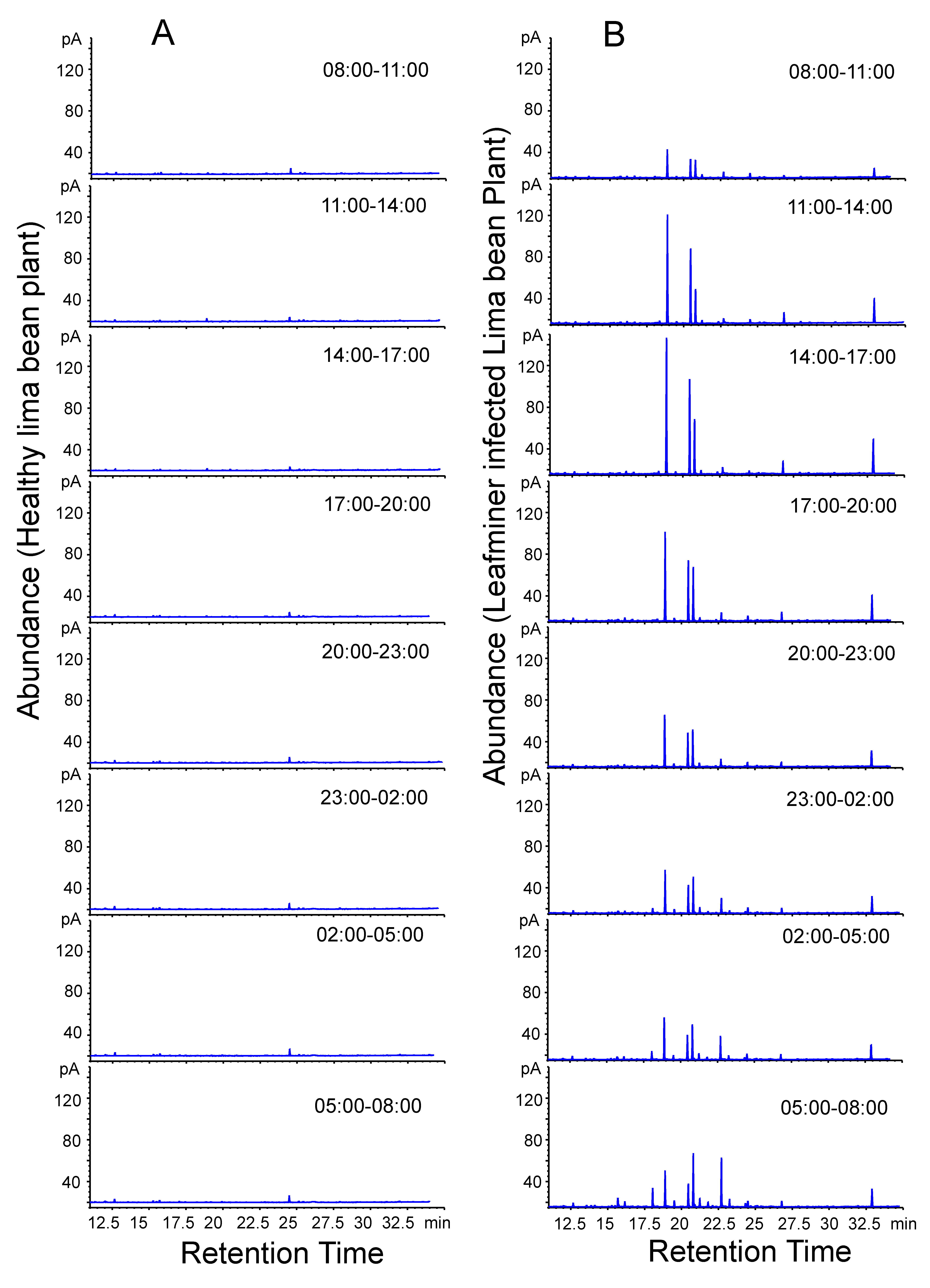

Supplement: Figure S1 — Headspace sample analysis with GC (7890A; Agilent Technologies, Inc., Santa Clara, CA, USA). (A), Headspace sample of healthy lima bean plant. (B), Headspace sample of 2 instars leafminer infected lima bean plant. Time intervals in the figure indicate the collection time interval of the headspace sample. (0.59 MB TIF) [file pone.0011064.s001.tif]
