# Supplementary material for: Functional Synchronization of Biological Rhythms in a Tritrophic System
Source: PLoS One. 2010 Jun 10;5(6):e11064. doi: 10.1371/journal.pone.0011064 (PMC2883855; doi:10.1371/journal.pone.0011064)
Supplement: Figure S2 — Diurnal rhythms of absolute amount of fourteen detected volatiles under three conditions (mean ± SE, n = 6). The bar under the x axis in each figure refers to the photoperiodic cycle; the black part refers to the dark phase. DMNT: (3E)-4,8-dimethyl-1,3,7-nonatriene; TMTT: (3E,7E)-4,8,12-trimethyl-1,3,7,11-tridecatetraene. (2.90 MB PDF) [file pone.0011064.s002.pdf]

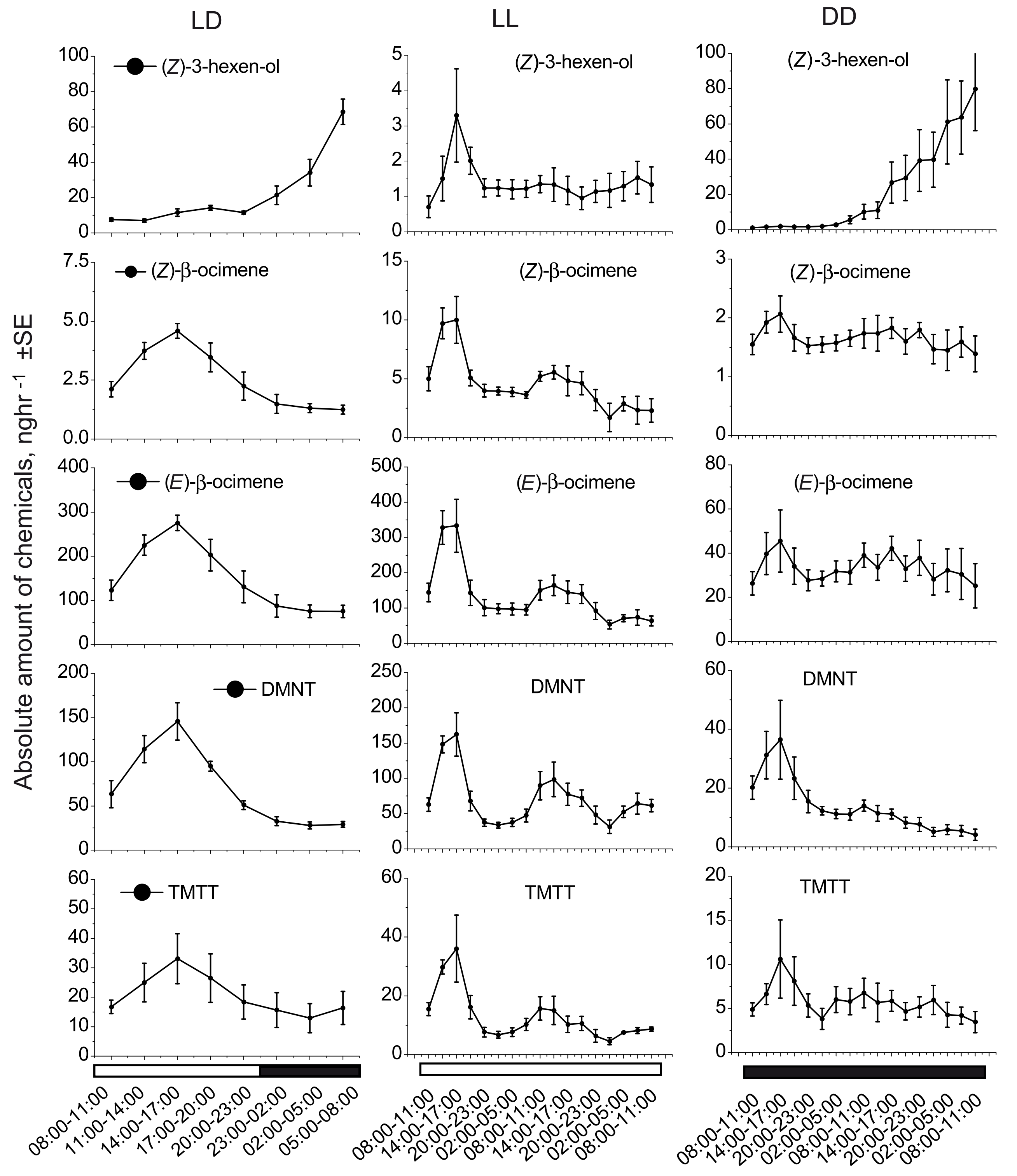

LD
LL
DD
(Z)-3-hexen-ol
(Z)-β-ocimene
(E)-β-ocimene
DMNT
TMTT
Absolute amount of chemicals, nghr -1 ±SE
08:00-11:00
11:00-14:00
14:00-17:00
17:00-20:00
20:00-23:00
23:00-02:00
02:00-05:00
05:00-08:00
08:00-11:00
14:00-17:00
20:00-23:00
02:00-05:00
08:00-11:00
14:00-17:00
20:00-23:00
02:00-05:00
08:00-11:00

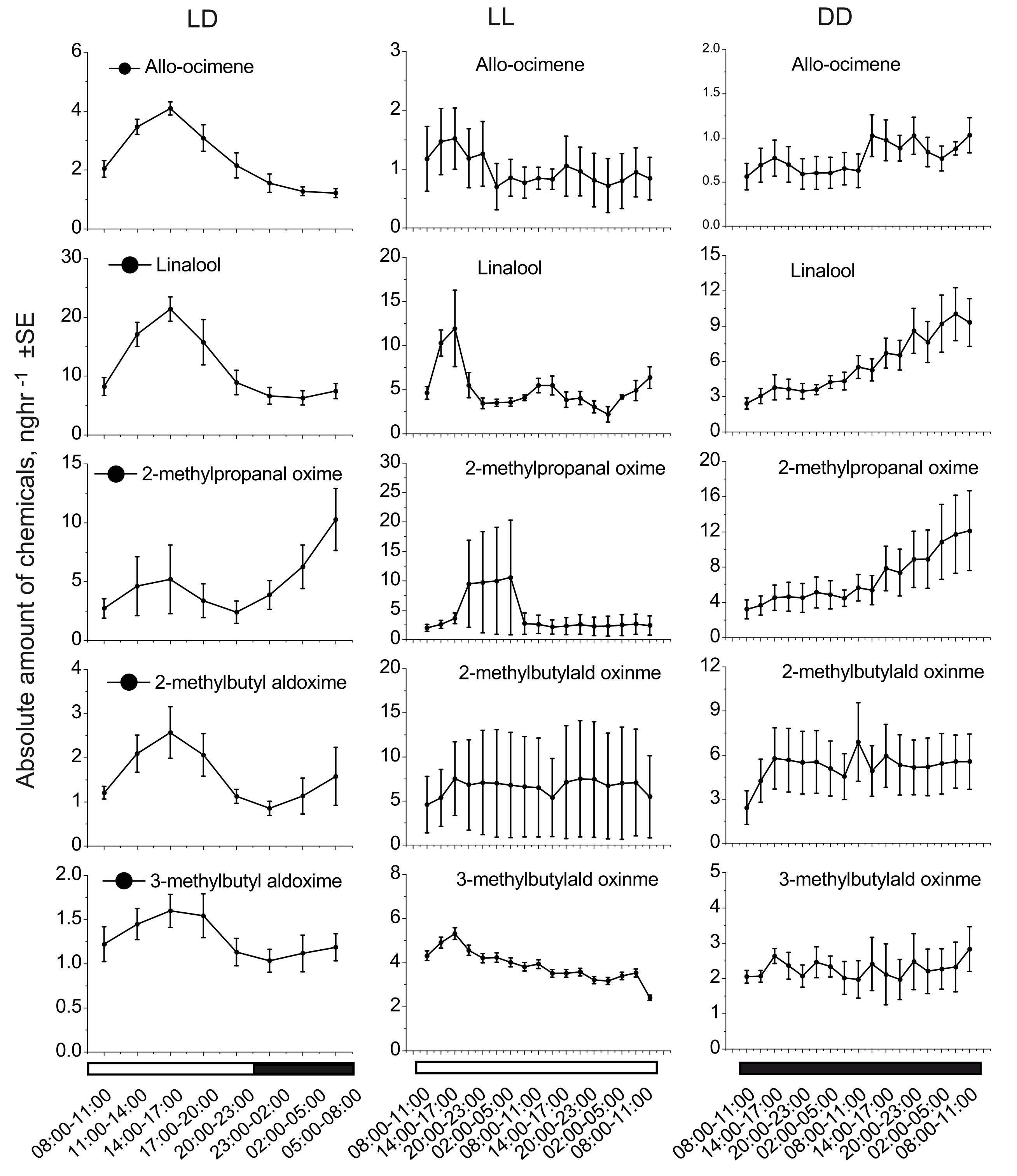

LD
LL
DD
Allo-ocimene
Linalool
2-methylpropanal oxime
2-methylbutyl aldoxime
3-methylbutyl aldoxime
2-methylbutylald oxinme
3-methylbutylald oxinme
Absolute amount of chemicals, nghr -1 ±SE
08:00-11:00
11:00-14:00
14:00-17:00
17:00-20:00
20:00-23:00
23:00-02:00
02:00-05:00
05:00-08:00
20:00-23:00
02:00-05:00

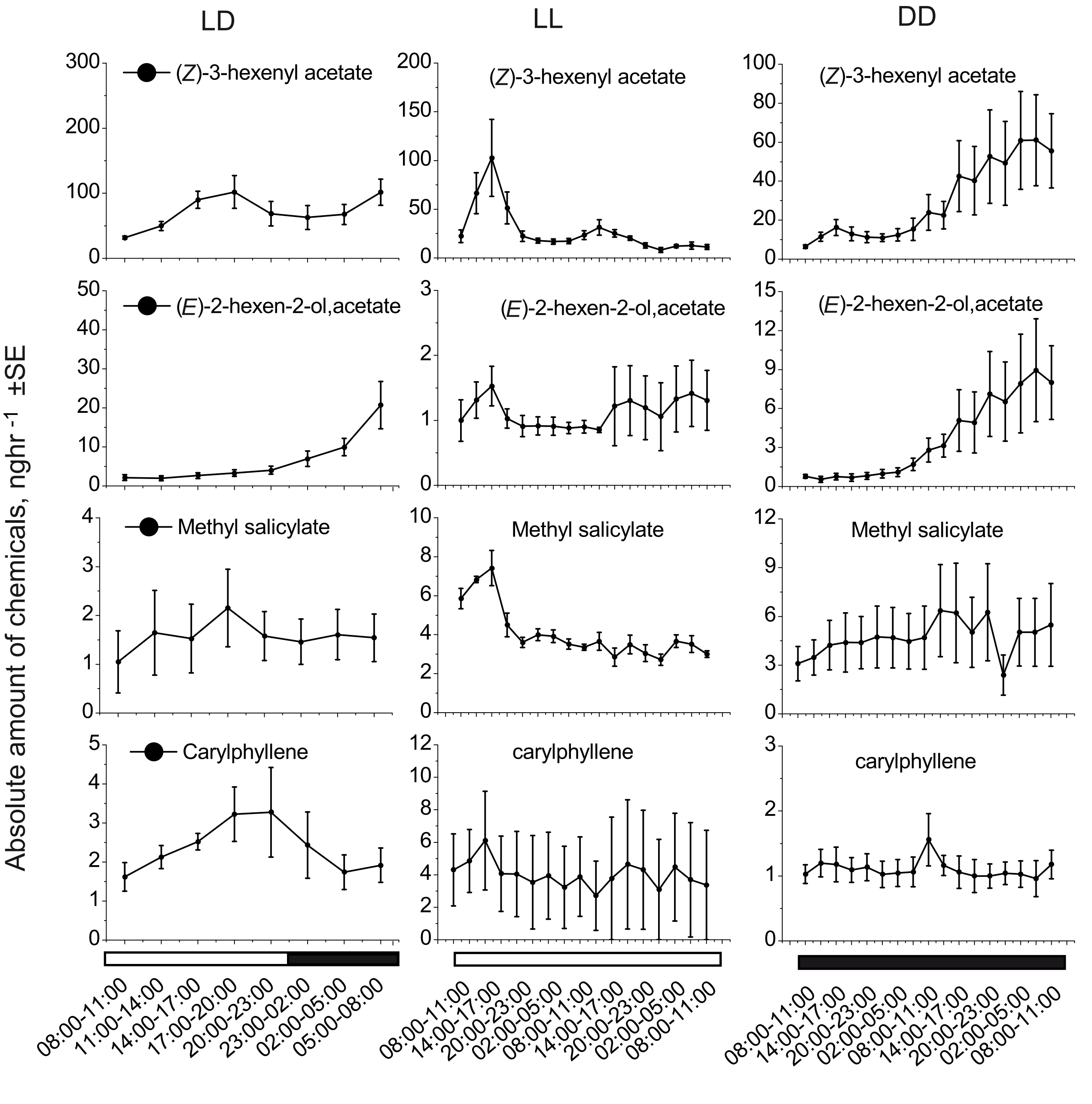

LD
LL
DD
(Z)-3-hexenyl acetate
(E)-2-hexen-2-ol,acetate
Methyl salicylate
Carylphyllene
carylphyllene
Absolute amount of chemicals, nghr -1 ±SE
08:00-11:00
11:00-14:00
14:00-17:00
17:00-20:00
20:00-23:00
23:00-02:00
02:00-05:00
05:00-08:00
14:00-17:00
20:00-23:00
02:00-05:00
08:00-11:00
