# Supplementary material for: Functional Synchronization of Biological Rhythms in a Tritrophic System
Source: PLoS One. 2010 Jun 10;5(6):e11064. doi: 10.1371/journal.pone.0011064 (PMC2883855; doi:10.1371/journal.pone.0011064)
Supplement: Table S1 — The most significant correlations among leafminer feeding, inducible emission and parasitoid activity (Pearson correlation at 0.01 level). (0.07 MB DOC) [file pone.0011064.s003.doc]

**Table S**1. The most significant correlations among leafminer feeding, inducible emission and parasitoid activity (Pearson correlation at 0.01 level).

|  | LF vs Ind-Vol | |  | Ind-Vol vs PE | |  | Ind-Vol vs PL | |  | Ind-Vol vs PO | |
| --- | --- | --- | --- | --- | --- | --- | --- | --- | --- | --- | --- |
|  |  | correlation |  |  | correlation |  |  | correlation |  |  | correlation |
| LD | LF vs Oci.1 | 0.849** |  | Hex vs PE | 0.787* |  | Oci.1 vs PL | 0.837** |  | Oci.1 vs PO | 0.849** |
|  | LF vs Oci.2 | 0.849** |  | P-Oxi vs PE | 0.945** |  | TMTT vs PL | 0.858** |  | Oci.2 vsPO | 0.837** |
|  | LF vs DMNT | 0.846** |  |  |  |  |  |  |  | TMTTvs PO | 0.875** |
|  | LF vs All-O | 0.868** |  |  |  |  |  |  |  | B-Oxi vs PO | 0.862** |
|  |  |  |  |  |  |  |  |  |  |  |  |
| LL | LF vs DMNT | 0.641** |  |  |  |  |  |  |  | Oci.2 vs PO | 0.643** |
|  |  |  |  |  |  |  |  |  |  | Hex3-A vs PO | 0.631** |
|  |  |  |  |  |  |  |  |  |  |  |  |
| DD |  |  |  |  |  |  | All-O vs PL | 0.711** |  |  |  |

Oci.1: (*Z*)-*β*-ocimene

Oci.2: (*E*)-*β*-ocimene

DMNT: (3*E*)-4,8-dimethyl-1,3,7–nonatriene

TMTT: (3*E*,7*E*)-4,8,12-trimethyl-1,3,7,11-tridecatetraene

All-O: All-ocimene

Hex3-A: (*Z*)-3-hexen-ol, acetate

Hex2-A: (*E*)-2-hexen-ol, acetate

Hex: (*Z*)-3-hexen-ol

P-Oxi: 2-methylpropanal oxime

B2-Oxi: 2-methylbutanal oxime

B3-Oxi: methylbutanal oxime

CPL: *β*-caryophellene

MeSA: methyl salicylate

LF: leafminer larval feeding

PE: parasitoid emergence

PO: parasitoid oviposition

PL: parasitoid locomotion
