# Supplementary material for: Functional Synchronization of Biological Rhythms in a Tritrophic System
Source: PLoS One. 2010 Jun 10;5(6):e11064. doi: 10.1371/journal.pone.0011064 (PMC2883855; doi:10.1371/journal.pone.0011064)
Supplement: Table S2 — Pearson Correlation analysis of the tritrophic interaction rhythms under LD (L∶D = 15∶9) cycle (Pearson correlation at 0.01 level). (0.12 MB DOC) [file pone.0011064.s004.doc]

**Table S2 Pearson Correlation analysis of the tritrophic interaction rhythms under LD (L:D=15:9) cycle (Pearson correlation at 0.01 level).**

|  | Oci.1 | Oci.2 | DMNT | TMTT | All-O | Linalool | Hex3-A | Hex2-A | Hex | P-Oxi | B2-Oxi | B3-Oxi | CPL | MeSA | LF | PE | PO | PL |
| --- | --- | --- | --- | --- | --- | --- | --- | --- | --- | --- | --- | --- | --- | --- | --- | --- | --- | --- |
| Oci.1 | 1 | 0.999** | 0.991** | 0.971** | 0.998** | 0.985** | 0.151 | -0.656 | -0.612 | -0.316 | 0.885** | 0.929** | 0.389 | 0.329 | 0.849** | -0.183 | 0.849** | 0.837** |
| Oci.2 |  | 1 | 0.993** | 0.972** | 0.998** | 0.988** | 0.152 | -0.642 | -0.598 | -0.295 | 0.892** | 0.929** | 0.374 | 0.32 | 0.849** | -0.178 | 0.837** | 0.825 |
| DMNT |  |  | 1 | 0.961** | 0.990** | 0.982** | 0.102 | -0.63 | -0.584 | -0.262 | 0.898** | 0.93** | 0.27 | 0.235 | 0.846** | -0.085 | 0.814 | 0.781 |
| TMTT |  |  |  | 1 | 0.962** | 0.984** | 0.352 | -0.499 | -0.45 | -0.17 | 0.927** | 0.941** | 0.434 | 0.387 | 0.743 | -0.202 | 0.875** | 0.858** |
| All-O |  |  |  |  | 1 | 0.978** | 0.108 | -0.679 | -0.639 | -0.342 | 0.866** | 0.911** | 0.384 | 0.3 | 0.868** | -0.178 | 0.829 | 0.82 |
| Linalool |  |  |  |  |  | 1 | 0.268 | -0.522 | -0.472 | -0.152 | 0.945** | 0.953** | 0.341 | 0.381 | 0.786 | -0.198 | 0.817 | 0.803 |
| Hex3-A |  |  |  |  |  |  | 1 | 0.47 | 0.513 | 0.506 | 0.438 | 0.347 | 0.451 | 0.686 | -0.226 | -0.398 | 0.369 | 0.406 |
| Hex2-A |  |  |  |  |  |  |  | 1 | 0.996** | 0.892** | -0.253 | -0.441 | -0.344 | -0.006 | -0.731 | 0.141 | -0.57 | -0.553 |
| Hex |  |  |  |  |  |  |  |  | 1 | 0.913** | -0.19 | -0.376 | -0.348 | 0.032 | -0.717 | 0.141 | -0.517 | -0.508 |
| P-Oxi |  |  |  |  |  |  |  |  |  | 1 | 0.138 | -0.087 | -0.46 | 0.039 | -0.475 | 0.153 | -0.381 | -0.395 |
| B2-Oxi |  |  |  |  |  |  |  |  |  |  | 1 | 0.963** | 0.204 | 0.419 | 0.655 | -0.074 | 0.754 | 0.732 |
| B3-Oxi |  |  |  |  |  |  |  |  |  |  |  | 1 | 0.289 | 0.456 | 0.73 | -0.07 | 0.862** | 0.83 |
| CPL |  |  |  |  |  |  |  |  |  |  |  |  | 1 | 0.641 | 0.289 | -0.622 | 0.577 | 0.706 |
| MeSA |  |  |  |  |  |  |  |  |  |  |  |  |  | 1 | 0.116 | -0.67 | 0.418 | 0.52 |
| LF |  |  |  |  |  |  |  |  |  |  |  |  |  |  | 1 | 0.088 | 0.626 | 0.662 |
| PE |  |  |  |  |  |  |  |  |  |  |  |  |  |  |  | 1 | -0.148 | -0.244 |
| PO |  |  |  |  |  |  |  |  |  |  |  |  |  |  |  |  | 1 | 0.974** |
| PL |  |  |  |  |  |  |  |  |  |  |  |  |  |  |  |  |  | 1 |

Oci.1: (*Z*)-*β*-ocimene

Oci.2: (*E*)-*β*-ocimene

DMNT: (3*E*)-4,8-dimethyl-1,3,7–nonatriene

TMTT: (3*E*,7*E*)-4,8,12-trimethyl-1,3,7,11-tridecatetraene

All-O: All-ocimene

Hex3-A: (*Z*)-3-hexen-ol, acetate

Hex2-A: (*E*)-2-hexen-ol, acetate

Hex: (*Z*)-3-hexen-ol

P-Oxi: 2-methylpropanal oxime

B2-Oxi: 2-methylbutanal oxime

B3-Oxi: methylbutanal oxime

CPL: *β*-caryophellene

MeSA: methyl salicylate

LF: leafminer larval feeding

PE: parasitoid emergence

PO: parasitoid oviposition

PL: parasitoid locomotion
