# Supplementary material for: Functional Synchronization of Biological Rhythms in a Tritrophic System
Source: PLoS One. 2010 Jun 10;5(6):e11064. doi: 10.1371/journal.pone.0011064 (PMC2883855; doi:10.1371/journal.pone.0011064)
Supplement: Table S3 — Pearson Correlation analysis of the tritrophic interaction rhythms under constant light (LL) cycle (Pearson correlation at 0.01 level). (0.12 MB DOC) [file pone.0011064.s005.doc]

**Table S3 Pearson Correlation analysis of the tritrophic interaction rhythms under constant light (LL) cycle (Pearson correlation at 0.01 level).**

|  | Oci.1 | Oci.2 | DMNT | TMTT | All-O | Linalool | Hex3-A | Hex2-A | Hex | P-Oxi | B2-Oxi | B3-Oxi | CPL | MeSA | LF | PE | PO | PL |
| --- | --- | --- | --- | --- | --- | --- | --- | --- | --- | --- | --- | --- | --- | --- | --- | --- | --- | --- |
| Oci.1 | 1 | 0.991** | 0.904** | 0.953** | 0.801** | 0.856** | 0.919** | 0.252 | 0.599 | -0.038 | -0.144 | 0.809** | 0.668** | 0.861** | 0.558 | -0.252 | 0.643** | 0.171 |
| Oci.2 |  | 1 | 0.936** | 0.964** | 0.809** | 0.884** | 0.924** | 0.341 | 0.617** | -0.123 | -0.143 | 0.767** | 0.677** | 0.859** | 0.599 | -0.229 | 0.587 | 0.132 |
| DMNT |  |  | 1 | 0.945** | 0.714** | 0.93** | 0.868** | 0.489 | 0.657** | -0.346 | -0.17 | 0.562 | 0.599 | 0.743** | 0.641** | -0.082 | 0.362 | 0.132 |
| TMTT |  |  |  | 1 | 0.805** | 0.945** | 0.95** | 0.377 | 0.719** | -0.159 | -0.192 | 0.753** | 0.68** | 0.89** | 0.551 | -0.101 | 0.511 | 0.068 |
| All-O |  |  |  |  | 1 | 0.741** | 0.824** | 0.398 | 0.559 | 0.066 | -0.097 | 0.751** | 0.73** | 0.811** | 0.522 | -0.21 | 0.557 | -0.05 |
| Linalool |  |  |  |  |  | 1 | 0.892** | 0.524 | 0.757** | -0.18 | -0.197 | 0.596 | 0.639** | 0.825** | 0.574 | -0.002 | 0.361 | 0.028 |
| Hex3-A |  |  |  |  |  |  | 1 | 0.381 | 0.843** | 0.04 | 0.021 | 0.798** | 0.706** | 0.851** | 0.428 | -0.264 | 0.631** | 0.249 |
| Hex2-A |  |  |  |  |  |  |  | 1 | 0.463 | -0.429 | 0.257 | 0.009 | 0.622** | 0.321 | 0.21 | -0.012 | -0.234 | -0.119 |
| Hex |  |  |  |  |  |  |  |  | 1 | 0.106 | 0.31 | 0.559 | 0.578 | 0.582 | 0.1 | -0.13 | 0.454 | 0.398 |
| P-Oxi |  |  |  |  |  |  |  |  |  | 1 | 0.27 | 0.367 | -0.008 | 0.03 | -0.317 | -0.329 | 0.58 | 0.323 |
| B2-Oxi |  |  |  |  |  |  |  |  |  |  | 1 | 0.028 | 0.285 | -0.263 | -0.631** | -0.254 | 0.199 | 0.641** |
| B3-Oxi |  |  |  |  |  |  |  |  |  |  |  | 1 | 0.633 | 0.843** | 0.213 | -0.192 | 0.783 | 0.136 |
| CPL |  |  |  |  |  |  |  |  |  |  |  |  | 1 | 0.731** | 0.12 | -0.292 | 0.375 | 0.005 |
| MeSA |  |  |  |  |  |  |  |  |  |  |  |  |  | 1 | 0.484 | -0.098 | 0.482 | -0.2 |
| LF |  |  |  |  |  |  |  |  |  |  |  |  |  |  | 1 | -0.106 | 0.103 | -0.292 |
| PE |  |  |  |  |  |  |  |  |  |  |  |  |  |  |  | 1 | -0.347 | -0.333 |
| PO |  |  |  |  |  |  |  |  |  |  |  |  |  |  |  |  | 1 | 0.463 |
| PL |  |  |  |  |  |  |  |  |  |  |  |  |  |  |  |  |  | 1 |

Oci.1: (Z)-β-ocimene

Oci.2: (E)-β-ocimene

DMNT: (3E)-4,8-dimethyl-1,3,7–nonatriene

TMTT: (3E,7E)-4,8,12-trimethyl-1,3,7,11-tridecatetraene

All-O: All-ocimene

Hex3-A: (Z)-3-hexen-ol, acetate

Hex2-A: (E)-2-hexen-ol, acetate

Hex: (Z)-3-hexen-ol

P-Oxi: 2-methylpropanal oxime

B2-Oxi: 2-methylbutanal oxime

B3-Oxi: methylbutanal oxime

CPL: β-caryophellene

MeSA: methyl salicylate

LF: leafminer larval feeding

PE: parasitoid emergence

PO: parasitoid oviposition

PL: parasitoid locomotion
