# Supplementary material for: Functional Synchronization of Biological Rhythms in a Tritrophic System
Source: PLoS One. 2010 Jun 10;5(6):e11064. doi: 10.1371/journal.pone.0011064 (PMC2883855; doi:10.1371/journal.pone.0011064)
Supplement: Table S4 — Pearson Correlation analysis of the tritrophic interaction rhythms under constant dark (DD) cycle (Pearson correlation at 0.01 level). (0.12 MB DOC) [file pone.0011064.s006.doc]

**Table S4 Pearson Correlation analysis of the tritrophic interaction rhythms under constant dark (DD) cycle (Pearson correlation at 0.01 level).**

|  | Oci.1 | Oci.2 | DMNT | TMTT | All-O | Linalool | Hex3-A | Hex2-A | Hex | P-Oxi | B2-Oxi | B3-Oxi | | CPL | MeSA | LF | PE | PO | PL | |
| --- | --- | --- | --- | --- | --- | --- | --- | --- | --- | --- | --- | --- | --- | --- | --- | --- | --- | --- | --- | --- |
| Oci.1 | 1 | 0.916** | 0.719** | 0.743** | 0.084 | -0.34 | -0.31 | -0.375 | -0.463 | -0.432 | 0.109 | | -0.024 | 0.136 | 0.298 | 0.478 | -0.173 | 0 | | 0.048 |
| Oci.2 |  | 1 | 0.547 | 0.706** | 0.169 | -0.11 | -0.07 | -0.166 | -0.269 | -0.211 | 0.362 | | -0.046 | 0.245 | 0.35 | 0.364 | -0.25 | 0 | | 0.195 |
| DMNT |  |  | 1 | 0.787** | -0.41 | -0.73** | -0.68** | -0.734** | -0.673** | -0.706** | -0.179 | | -0.026 | -0.363 | 0.302 | 0.451 | -0.232 | 0 | | -0.287 |
| TMTT |  |  |  | 1 | -0.19 | -0.46 | -0.44 | -0.518** | -0.541** | -0.512** | 0.182 | | 0.03 | -0.242 | 0.366 | 0.405 | -0.254 | 0 | | -0.101 |
| All-O |  |  |  |  | 1 | 0.712** | 0.721** | 0.717** | 0.656** | 0.668** | 0.24 | | 0.447 | 0.623** | -0.18 | -0.318 | -0.089 | 0 | | 0.711** |
| Linalool |  |  |  |  |  | 1 | 0.986** | 0.989** | 0.951** | 0.978** | 0.37 | | 0.313 | 0.414 | -0.214 | -0.536 | 0.215 | 0 | | 0.508 |
| Hex3-A |  |  |  |  |  |  | 1 | 0.99** | 0.946** | 0.968** | 0.325 | | 0.259 | 0.356 | -0.261 | -0.472 | 0.133 | 0 | | 0.559 |
| Hex2-A |  |  |  |  |  |  |  | 1 | 0.959** | 0.972** | 0.264 | | 0.264 | 0.36 | -0.27 | -0.502 | 0.211 | 0 | | 0.516 |
| Hex |  |  |  |  |  |  |  |  | 1 | 0.983** | 0.231 | | 0.384 | 0.3 | -0.237 | -0.425 | 0.248 | 0 | | 0.475 |
| P-Oxi |  |  |  |  |  |  |  |  |  | 1 | 0.355 | | 0.389 | 0.356 | -0.25 | -0.502 | 0.195 | 0 | | 0.522 |
| B2-Oxi |  |  |  |  |  |  |  |  |  |  | 1 | | 0.208 | 0.404 | 0.415 | -0.352 | -0.174 | 0 | | 0.374 |
| B3-Oxi |  |  |  |  |  |  |  |  |  |  |  | | 1 | 0.328 | -0.118 | -0.438 | -0.12 | 0 | | 0.186 |
| CPL |  |  |  |  |  |  |  |  |  |  |  | |  | 1 | -0.029 | -0.287 | -0.019 | 0 | | 0.399 |
| MeSA |  |  |  |  |  |  |  |  |  |  |  | |  |  | 1 | 0.32 | 0.037 | 0 | | -0.236 |
| LF |  |  |  |  |  |  |  |  |  |  |  | |  |  |  | 1 | -0.229 | 0 | | -0.196 |
| PE |  |  |  |  |  |  |  |  |  |  |  | |  |  |  |  | 1 | 0 | | -0.257 |
| PO |  |  |  |  |  |  |  |  |  |  |  | |  |  |  |  |  | 1 | | 0 |
| PL |  |  |  |  |  |  |  |  |  |  |  | |  |  |  |  |  |  | | 1 |

Oci.1: (*Z*)-*β*-ocimene

Oci.2: (*E*)-*β*-ocimene

DMNT: (3*E*)-4,8-dimethyl-1,3,7–nonatriene

TMTT: (3*E*,7*E*)-4,8,12-trimethyl-1,3,7,11-tridecatetraene

All-O: All-ocimene

Hex3-A: (*Z*)-3-hexen-ol, acetate

Hex2-A: (*E*)-2-hexen-ol, acetate

Hex: (*Z*)-3-hexen-ol

P-Oxi: 2-methylpropanal oxime

B2-Oxi: 2-methylbutanal oxime

B3-Oxi: methylbutanal oxime

CPL: *β*-caryophellene

MeSA: methyl salicylate

LF: leafminer larval feeding

PE: parasitoid emergence

PO: parasitoid oviposition

PL: parasitoid locomotion
